# Evidence That Putrescine Modulates the Higher Plant Photosynthetic Proton Circuit

**DOI:** 10.1371/journal.pone.0029864

**Published:** 2012-01-12

**Authors:** Nikolaos E. Ioannidis, Jeffrey A. Cruz, Kiriakos Kotzabasis, David M. Kramer

**Affiliations:** 1 Department of Biology, University of Crete, Heraklion, Crete, Greece; 2 Institute of Biological Chemistry, Washington State University, Pullman, Washington, United States of America; 3 Department of Biochemistry and Molecular Biology and DOE-Plant Research Laboratory, Michigan State University, East Lansing, Michigan, United States of America; University of California – Davis, United States of America

## Abstract

The light reactions of photosynthesis store energy in the form of an electrochemical gradient of protons, or proton motive force (*pmf*), comprised of electrical (Δψ) and osmotic (ΔpH) components. Both components can drive the synthesis of ATP at the chloroplast ATP synthase, but the ΔpH component also plays a key role in regulating photosynthesis, down-regulating the efficiency of light capture by photosynthetic antennae via the q_E_ mechanism, and governing electron transfer at the cytochrome b_6_f complex. Differential partitioning of *pmf* into ΔpH and Δψ has been observed under environmental stresses and proposed as a mechanism for fine-tuning photosynthetic regulation, but the mechanism of this tuning is unknown. We show here that putrescine can alter the partitioning of *pmf* both *in vivo* (in *Arabidopsis* mutant lines and in *Nicotiana* wild type) and *in vitro*, suggesting that the endogenous titer of weak bases such as putrescine represents an unrecognized mechanism for regulating photosynthetic responses to the environment.

## Introduction

The light-driven transthylakoid proton motive force (*pmf*) plays several essential roles in photosynthesis [1]. Both the ΔpH (osmotic) and Δψ (electric) components of *pmf* contribute to ATP synthesis at the CF_O_-CF_1_ ATP synthase, in a thermodynamically equivalent fashion [2], but the ΔpH component of *pmf* is also a key signal for initiating photoprotection of the photosynthetic reaction centers through energy-dependent non-photochemical quenching (q_E_), a process that dissipates excess absorbed light energy as heat, thus protecting the photosynthetic apparatus from photodamage [3]–[5]. Acidification of the lumen also controls photosynthetic electron transfer by slowing the rate of plastoquinol oxidation at the cytochrome b_6_f complex [6], [7], preventing the accumulation of highly reducing species within photosystem I [8].

Differential partitioning of the thylakoid *pmf* into ΔpH and Δψ components has been observed in thylakoids [9] and in intact leaves [10] and was proposed to constitute an important fine-tuning mechanism for photosynthesis [11]. Under optimal conditions, when down-regulation is not needed, a large fraction of *pmf* can be stored as Δψ, leading to moderate lumen pH and low q_E_, even at high *pmf* (and thus high rates of ATP synthesis). In contrast, under environmental stresses—e.g., high light, low CO_2_/O_2_, when photoprotection is advantageous—*pmf* can be predominantly stored as **Δ**pH, maximizing lumen acidification for a given *pmf*.

The mechanism by which thylakoid *pmf* is partitioned into Δψ and ΔpH remains unclear, but *in vitro* experiments and modeling have established that at least three factors are critical [9], [11] : 1) the capacitance of the thylakoid membrane, which determines the Δψ generated for the transfer of a charge across the membrane; 2) the proton-buffering capacity of the lumen, which determines the relationship between translocated protons and changes in lumen pH; and 3) the ionic composition of the stroma and lumen, which determines the degree to which movements of counterions can dissipate the Δψ component. Of these three, ionic balance appears to be the most likely to account for the observed short-term changes in *pmf* partitioning in response to environmental changes, because thylakoid membrane capacitance and lumen buffering capacity are thought to change only slowly, whereas plastids can rapidly alter the ionic balance by regulating ion channels or ion pumps [9], [11].

More recently, Ioannidis et al. [12] proposed an alternative (but non-exclusive) hypothesis—the ‘biological weak base” (BWB) hypothesis—for Δψ/ΔpH control. This model involves biological weak bases, such as polyamines, which occur normally in chloroplasts and may act as ‘permeant buffers’, specifically dissipating the ΔpH component and thus favoring Δψ. Because the titer of these weak bases can be regulated by the organism (by synthesis, degradation, transport, covalent binding to proteins, and phenolics), this mechanism may constitute a means to adjust the ΔpH/Δψ ratio in the short (seconds) and long term (hours to days).

Putrescine (Put) is a diamine [NH_2_(CH_2_)_4_NH_2_], which, along with spermidine and spermine, constitute the major polyamines in plants. Polyamines are important or even essential for many cellular processes, such as cell growth and stress tolerance [13]–[15]. Although the metabolism of polyamines is well understood, their mode of action is ill defined (for reviews see refs [16] and [17]). The proposed mechanism for putrescine action is similar to that by which amines dissipate the ΔpH component of *pmf* in isolated thylakoids [18]. Under physiological pH, the protonated forms of amines prevail but are in equilibrium with a small concentration of free base, which can permeate the membrane (see Figure S1). Because these forms are positively charged, they cannot readily cross the thylakoid membrane. Acidification of the lumen will displace the equilibrium toward the charged forms, in turn allowing diffusion of more free forms across the thylakoid membrane into the lumen. Net transfer of weak bases from stroma to lumen and conversion to the protonated forms dissipates (buffers) ΔpH, but builds up a gradient of charged bases. Because the process is electroneutral with respect to the thylakoid membrane, weak bases do not dissipate (or augment) the Δψ component of *pmf*. However, weak bases in the presence of high concentrations of counterions, which are permeable through ion channels, can dissipate both the Δψ and ΔpH components of *pmf* [18]. It is important to note that the concentrations of permeable ions in chloroplasts *in vivo* is likely to be small [2], [9], so that weak bases should primarily affect the ΔpH component of *pmf*.

## Results and Discussion

**Figure 1:**
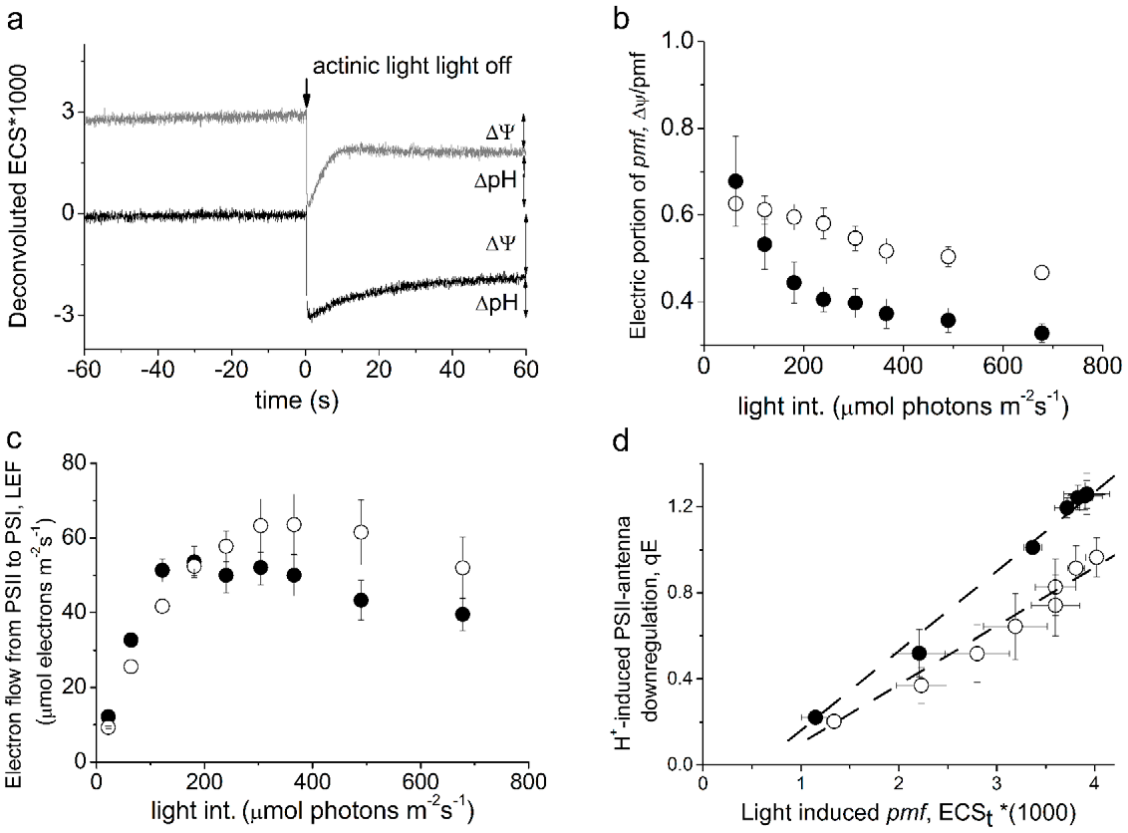
Regulation of the electric field component (Δψ) of *pmf*. Panel A shows typical deconvoluted traces obtained from intact tobacco leaves incubated with water (gray) or reinfiltrated with 3 mM putrescine (Put; black). Steady-state *pmf* was probed at 8 different light intensities. Leaves with elevated putrescine (open symbols) show up to 40% higher Δψ/*pmf* than the corresponding controls (closed symbols) (Panel B). Buffering of the thylakoid lumen by elevated putrescine levels allows more efficient electron transfer at higher light intensity in comparison to low putrescine levels (Panel C). D. Energy-dependent antenna down-regulation (q_E_) as a function of the ΔpH component of the light-induced *pmf*. The linear fit for control has a slope of 0.369 (R^2^ = 0.998) and for putrescine treated a slope of 0.272 (R^2^ = 0.992), showing that putrescine supply decreases sensitivity of qE to ECSt by about 27%. All single points are means from 4 independent experiments performed with intact tobacco leaves and bars denote standard error.

As a first test of the BWB hypothesis *in vivo*, we assessed the fractions of *pmf* stored as Δψ and ΔpH using *in vivo* spectroscopic techniques [7], [10], [19] in leaves depleted of or re-infiltrated with putrescine. We found that putrescine is highly mobile and readily diffuses into (Figure S2) and out of (Figure S3) leaves through cut petioles placed in water solutions. In figure S3 we report a decrease of the endogenous titer of putrescine. It is plausible to assume that at least in part this is due to loss of putrescine from the cut petiole. This leakage of endogenous putrescine from the leaf to the water is in line with the increase of putrescine titer of the solution from 0 µM to about 3.2 µM. Using cut tobacco leaves, which are stable in such solutions for long periods (e.g., Figure S3 which shows that photosystem II is stable in tobacco leaves for 48 h), we were able to deplete and replete putrescine levels, as confirmed by HPLC analysis [20]. Incubation for ∼15 h after leaf detachment led to a 47% decrease in putrescine titer, whereas feeding putrescine with 6 mM increased putrescine 4–5 times (Figures S2 and S3). After 16 h of incubation of the leaf petiole in a 3 mM putrescine solution, the putrescine titer was increased at the tip of the leaf about 2-fold. Figure 1a shows representative kinetic traces of the decay of the electrochromic shift (ECS) signal around 520 nm upon a light-dark transition, in control, putrescine-depleted, and re-infiltrated with putrescine samples. As described in ref. [11], the extent of the rapid phase reflects the total light-driven *pmf*, whereas the slowly recovering phase is attributed specifically to the **Δ**pH portion. Deconvolution, as indicated, yields an estimate of the fraction of *pmf* stored as **Δ**pH and **Δ**ψ (Figure 1a). We observed a clear increase, by ∼36%, in the **Δ**pH-related phase (or decrease of **Δ**ψ) upon partial depletion of putrescine, which was reversed by putrescine infiltration (Figure 1b). Complementary photosynthesis measurements were also consistent with a higher **Δ**pH upon depletion of putrescine. In comparison to control leaves, infiltrated with water, those with elevated putrescine showed decreased q_E_ responses and higher rates of linear electron transfer (Figure 1c and d). The response of q_E_ to the total light-induced *pmf*, estimated by ECS decay, was lower when leaf putrescine was elevated (Figure 1d), consistent with a smaller fraction of *pmf* stored as ΔpH (Figure 1b). Also, the sensitivity of q_E_ to light-induced **Δ**pH *in vivo* [as estimated by analyzing the decay kinetics of the ECS [9]] was found to be similar between treated and control leaves (Figure S4). Noteworthy is that putrescine at higher doses decrease even more the sensitivity of qE to light induced *pmf* by increasing **Δ**ψ and this effect is reversed to a great extent upon transfer of the leaf petiole from the putrescine solution to distilled water (Figure S5).

**Figure 2:**
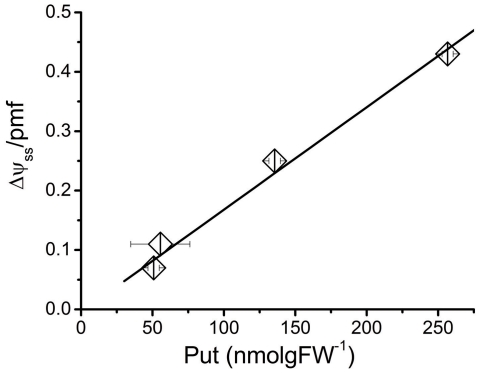
The dependence of Δψ/*pmf* in intact leaves on endogenous putrescine titer. Partitioning of *pmf* in excised tobacco leaves. Detachment of leaves and insertion of the petiole in distilled water leads to a gradual decrease of putrescine titer in the leaf, which in turn leads to a decrease in the Δψ fraction of *pmf*. Data correspond to the first two days after detachment (Fv/Fm decrease during this period was no more than 10%) and bars denote standard error (n = 2). Linear regression of putrescine titer versus Δψ is shown as a solid line with a slope of 0.00173 (R^2^ = 0.995).

**Figure 3:**
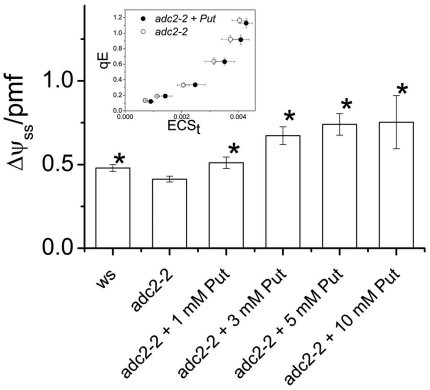
The Δψ/*pmf* in intact plants of *Arabidopsis*. The partitioning of *pmf in vivo* into the electric component (Δψ) for intact *Arabidopsis* leaves and the effect of putrescine. Low titer of putrescine (*adc2-2*) leads to lower Δψ/*pmf* in comparison to the wild type (ws). The exogenous supply of putrescine (in various doses from 1 mM up to 10 mM) leads to an increase of the fractionation of *pmf* to Δψ to values even higher than those of wild type. The ECS signal was measured and deconvoluted as described in Materials and Methods. Error bars denote standard error for n≥3 and the asterisk denotes statistically (t-test) significant, at the 0.05 level, difference to the *adc2-2* mean values (second column). Mean values were compared only to the adc2-2 which has the lowest putrescine titer. Inset: Energy-dependent antenna down-regulation (q_E_) as a function of the light-induced *pmf* (ECS_t_) for putrescine-treated *adc2-2* plants (closed circles) and controls in distilled water (open circles). A linear regression model was fitted to the data using log-transformed q_E_ values. The mean ECS_t_ response is significantly different between “putrescine fed *adc2-2*” and controls (*P* = 0.0047), across the range of the observed q_E_ values.

The above results and those from a time-course study (Figures 2 and S3) suggest that putrescine depletion induces an increase in the **Δ**pH component of thylakoid *pmf*, but do not determine whether this effect is direct or indirect. We thus tested for effects of mutants of *Arabidopsis thaliana* Columbia (*Arabidopsis*) deficient in putrescine synthesis. Plants completely lacking putrescine are not viable [21] and thus we used a mutant, *adc2-2* [21], which under our experimental conditions accumulated putrescine to about 40% wild-type levels (∼40 vs. ∼100 nmol g FW^−1^). Estimates of *pmf* partitioning using the decay of the ECS [11] gave evidence that moderate decreases in putrescine in *adc2-2* led to a ∼15% smaller fraction of *pmf* attributable to Δψ [or a larger fraction stored as **Δ**pH (Figure 3)]. The effect was reversed by feeding putrescine into leaf petioles, supporting a direct effect of putrescine on *pmf* partitioning rather than indirect biochemical or developmental effects. A larger portion of *pmf* stored as ΔpH in putrescine-deficient plants is also supported by a higher sensitivity of the photoprotective qE response, a process dependent on lumen acidification, to pmf as estimated by the extent of the light-induced ECS signal (Figure 3, inset). Further analysis of the ECS decay kinetics [22], showed that elevated putrescine had no discernable effect on the activity of the thylakoid ATP synthase (Figure S6).

**Figure 4:**
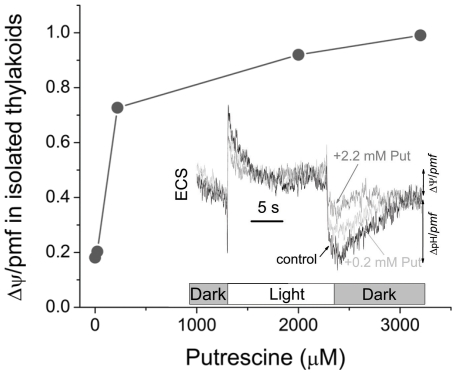
Dose course for the Δψ/*pmf* in isolated thylakoids as a function of putrescine concentration. Thylakoids isolated from spinach were pre-incubated in the dark in solution containing 0 mM, 0.02 mM, 0.22 mM, 2.2 mM, or 3.2 mM putrescine. ECS was measured during and after 16 s of actinic illumination. Selected deconvoluted traces are illustrated as an insert. Fractional storage of *pmf* as Δψ was estimated and plotted as a function of the putrescine concentration.

The above experiments show a positive relationship between putrescine titer in leaves, altered by depletion, infiltration, or mutation, and the fraction of *pmf* stored as Δψ, consistent with the BWB hypothesis. At pH ∼7.5 or lower, putrescine should predominantly be in its +2 state (pK_1_ = 10.5, pK_2_ = 9.04), and its effects have been attributed to its ability to bind anions, nucleic acids, and other negatively charged molecules or domains [15], [17], [23]. It is possible that putrescine affects *pmf* partitioning by scavenging anions that would otherwise permeate the thylakoid and dissipate Δψ. To test these possibilities, we assayed the effects of putrescine on Δψ/*pmf* using ECS assays [9], [24] in isolated spinach thylakoids in buffer with set ionic composition. The dependence of Δψ/*pmf* on putrescine concentration is shown in Figure 4 with selected ECS traces shown in the inset. Putrescine is a naturally occurring solute in chloroplasts but the endogenous pool is lost during isolation procedures, together with other stromal solutes. In the presence of 0 mM KCl and only a low dose of bivalent cations (i.e., 0.15 mM MgCl_2_) (solution of low ionic strength), the fraction of *pmf* stored as Δψ was about 0.2 for control, indicating that a significant decline occurred after isolation in comparison to the in vivo conditions (Δψ/*pmf*∼0.5) (Figure 4). This decline at least partly reflects altered ionic composition of the suspension buffer compared to chloroplasts in vivo [9], but could also reflect the loss of putrescine or other mobile buffers. Adding putrescine to the suspension buffer increased Δψ/*pmf* of thylakoids within a few seconds, indicating that putrescine can rapidly modulate *pmf* partitioning. Addition of 220 µM putrescine led to an increase in Δψ/*pmf* to ∼0.6, and putrescine concentrations above 2 mM led to Δψ/*pmf*>0.9 (Figure 4). Our deconvolution suffers to some extent by drifts and noise due to in vitro conditions. Thus the absolute values of Δψ/*pmf* caused by putrescine could be slightly different to those of figure 4 (∼15%). However, the effect of putrescine on thylakoidal energization is initially a rapid increase of Δψ and then at a second phase a plateau.

The fact that putrescine decreased ΔpH even in a solution with low concentration of scavangeable anions, supports the operation of the BWB mechanism, and is also consistent with the effects of exogenously added weak bases in photosynthetic prokaryotes [25] and thylakoids [26], [27] in which amines have been shown to be concentrated by more than 100-fold on the low pH side of an energized membrane, with concomitant effects on ΔpH. We would suggest that putrescine is a good compromise between balancing ΔpH and Δψ and avoiding secondary deleterious effects. In addition, putrescine levels in the cell is so finely tuned through multiple ways (synthesis through two highly regulated pathways, conversion from Spermidine, transport from other cell compartments or neighbour cells, release from conjugate with phenolics, binding to proteins etc [15], [16]) that one can not find easily other cell metabolites that can adjust their level so rapidly and accurately to meet the ever changing demand. So although ammonia and methylamine could act in a similar way to putrescine their titer in cells is lower than that of putrescine and thus their importance in the BWB mechanism should be lower.

Assuming that putrescine is evenly distributed in leaves, we estimate its cellular concentration in tobacco leaves at about 275 µM (based on measured value of 250 nmoles Put/g fresh weight and assuming 90% of leaf mass is water; see also, ref. [28]. When thylakoids were suspended in buffer containing this concentration of putrescine in thylakoids, we observed about 50% of *pmf* stored as Δψ and ΔpH (Figure 4), similar to what is observed in healthy, unstressed leaves [9], [11]. However, it is important to note that the partitioning of *pmf* is also expected to be influenced by ionic composition, with increasing counterion concentration dissipating Δψ [9].

Putrescine concentrations in leaves are increased during environmental stress, in part due to up-regulation of chloroplast arginine decarboxylase (ADC, EC 4.1.1.19) [29]–[31]. One may thus expect to see a shift in the partitioning of *pmf* into Δψ under environmental stress. However, at least under drought stress in wild watermelon [32], short-term high light exposure in Arabidopsis [7], or low CO_2_ and O_2_ in tobacco [10], the opposite was observed, with a pronounced increase in ΔpH/*pmf*. A reasonable explanation is that the initial increase in ΔpH/*pmf* is due to stress-induced changes in ionic composition [9], [11] and that putrescine may ameliorate these effects over the long term. At a mechanistic level, consistent with this view, Cruz et al. [9], showed that elevated lumen buffering capacity will increase the counterion chemical activity needed to dissipate the Δψ component of *pmf*, whereas Ioannidis et al. [12] showed that putrescine can overcome photosynthetic control and thus stimulate chemiosmotic ATP synthesis in thylakoids of higher plants [12].

In *Arabidopsis* grown under high salt stress, photosynthesis would likely need to operate under conditions where the ionic strength inside the plastid is high. In this case, *pmf* storage would be heavily biased toward ΔpH formation [9], [33]. Consequently, energy dissipation would be more easily and strongly induced at low and moderate light intensities, severely limiting the productivity and growth of the plant, even if water and CO_2_ were not limiting. Thus, the accumulation of putrescine observed in plants grown under high salt stress [34], [35] and particularly in *Arabidopsis* through *adc2* induction [36] could serve to increase the BWB effect, rebalancing *pmf* toward Δψ and optimizing the regulation of energy transduction. In line with this view, blocking this up-regulation of putrescine during salt stress, e.g., in the *adc-2-1* mutant of *Arabidopsis*, leads to increased sensitivity to salt stress, which is restored upon addition of putrescine [36], whereas over-expressing *adc* increased tolerance to drought [13].

We present evidence that putrescine plays a role in modulating *pmf* partitioning *in vivo* via the BWB mechanism, possibly operating as a part of the regulatory network of photosynthesis. Putrescine levels in the leaf are known to be regulated at several levels, including rates of synthesis, catabolism, conversion to spermidine, conjugation, intracellular or extracellular transport, gene expression, and/or allostery [15]. Thus, putrescine homeostasis could provide the plant with an independent mechanism for adapting the q_E_ response to *pmf*, optimizing the balance between energy transduction and dissipation under a variety of stress conditions.

## Materials and Methods

### Spectroscopy

We conducted time-resolved spectroscopic measurements for estimation of LEF, q_E_, ECS_t_, and Δψ/*pmf* at room temperature using wild-type (Wt) *Arabidopsis thaliana* (Wassilevskija ecotype; WS) plants, the low putrescine *adc2-2* mutant [21], and wild-type *Nicotiana tabacum* cv *Xanthi* as previously described [10]. More particularly, LEF was calculated for the following expression 0.84*PAR*(Fm′-Ft)/Fm′) where Fm′ is the maximal fluorescence value of a light adapted leaf after a saturating pulse (>7,000 µmol photons m^−2^s^−1^) and Ft is the level of fluorescence immediately before the saturating pulse. For estimates of *pmf* partitioning, the ECS was measured over longer dark intervals (60 sec) and deconvoluted as previously described [10], [11] using the following equation for tobacco: ECS_520_ = A_520_ − 0.5×A_535_ − 0.5×A_505_. For more precise deconvolution of ECS signals from *Arabidopsis*, we derived an equation from empirically determined, relative extinction coefficients at each wavelength, based on methods described previously [37], [38]: ECS_520_ = −(1.61*A_520_−0.61*A_505_−1.17A_535_).

### Determination of putrescine titer

Putrescine titer in leaves was estimated after benzoylation of the amines, separation in HPLC, and quantitation of the derivatives as previously described [20].

### In vitro experiments

Thylakoids were isolated from market spinach as previously described [9]. Freshly isolated thylakoids (10 µg/mL Chl) were treated with various doses of the amine salt in the dark and, after equilibration, were subjected to light for 16 s. The final volume was 3 mL of a working solution containing 0.125 mM tricine, pH 7.8, 0.15 mM MgCl_2_, 0.3 M sucrose, 10% ficoll. Photosynthetic proton uptake was supported by 30 µM PMS. Spectroscopic measurements were performed, as above, with the sample contained in a cuvette (10-mm path length). For thylakoid samples, a simple deconvolution yielded consistent results: ECS_520_ = A_520_ − 1.2×A_535_.

## Supporting Information

Figure S1 **Simplified scheme for the regulation of** ***pmf*** **partitioning by Putrescine.**
(DOC)

Figure S2 **Assay for putrescine uptake into tobacco leaves by HPLC.**
(DOC)

Figure S3 **Effects on Δψ/** ***pmf*** **and PSII photochemical efficiency of infiltration of leaves with water.**
(DOC)

Figure S4 **Effect of putrescine titre on the dependence of energy-dependent antenna down-regulation (q_E_) on the ΔpH component of light-induced** ***pmf*** **.**
(DOC)

Figure S5 **Energy-dependent antenna down-regulation (q_E_) as a function of total light-induced** ***pmf*** **.**
(DOC)

Figure S6 **Effects of putrescine titre on the apparent proton conductivity of the ATP synthase (g_H_^+^).**
(DOC)
