# Supplementary material for: Evidence That Putrescine Modulates the Higher Plant Photosynthetic Proton Circuit
Source: PLoS One. 2012 Jan 12;7(1):e29864. doi: 10.1371/journal.pone.0029864 (PMC3257247; doi:10.1371/journal.pone.0029864)
Supplement: Figure S1 — Simplified scheme for the regulation of pmf partitioning by Putrescine. (DOC) [file pone.0029864.s001.doc]

**Supporting Information**

| 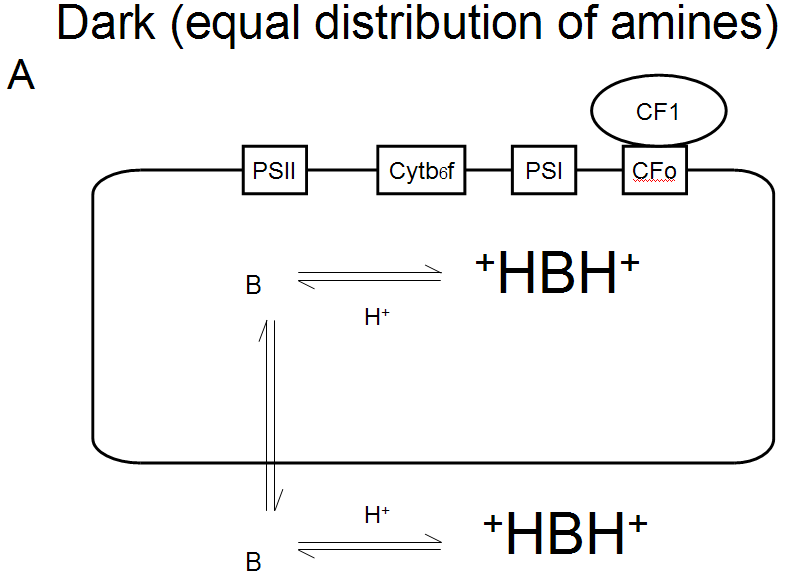 | 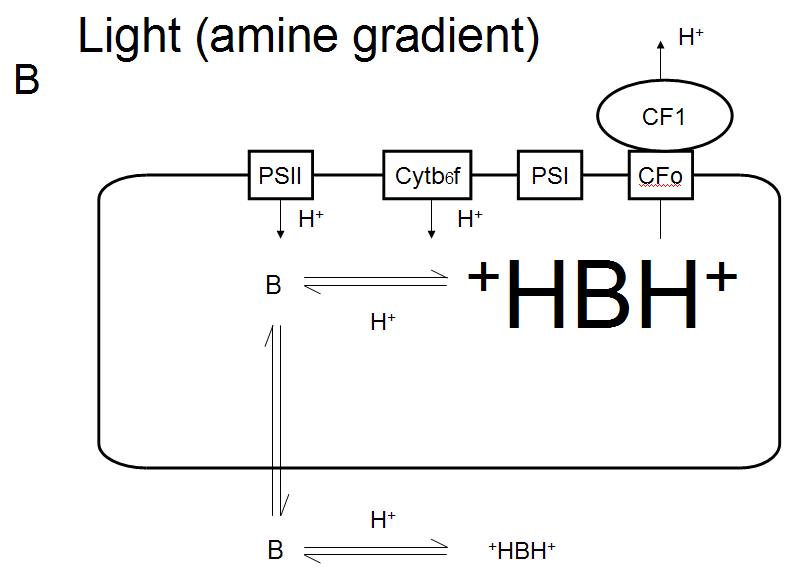 |
| --- | --- |

**Figure S1.** **Simplified scheme for the regulation of *pmf* partitioning by Put.**

A. During dark adaptation we assume that a minimal ΔpH occurs which allows ca. equal distribution of putrescine (bases with pKs higher than 7.5). Font size for bases (B) or protonated bases (^+^HBH^+^ ) correspond roughly to their concentration. B. Acidification of the thylakoid lumen by the light reactions favors the protonated (charged) over the deprotonated (neutral) forms of weak bases such as Put (pKs higher than 9), consuming protons in the lumen and allowing transfer of neutral Put from the stroma to the lumen. This results in buffering of protons in the lumen, depleting the ΔpH component of the *pmf*, while building up a gradient of protonated Put. Because the transfer of Put is electroneutral, it does not impact the Δψ component of pmf, thus increasing the ratio of Δψ/ΔpH. The resulting moderation of lumen acidity decreases acid-induced degradation of PSII, downregulation of electron transfer at the level of Cyt *b_6_f* and activation of the q_E_ response.
