# Supplementary material for: Evidence That Putrescine Modulates the Higher Plant Photosynthetic Proton Circuit
Source: PLoS One. 2012 Jan 12;7(1):e29864. doi: 10.1371/journal.pone.0029864 (PMC3257247; doi:10.1371/journal.pone.0029864)
Supplement: Figure S2 — Assay for putrescine uptake into tobacco leaves by HPLC. (DOC) [file pone.0029864.s002.doc]

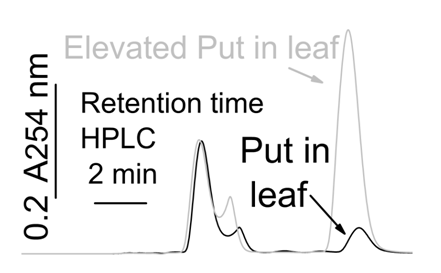


**Figure S2. Assay for Put uptake into tobacco leaves by HPLC.**  Uptake of Put by tobacco leaves was estimated by benzoyl-derivatives of Put separated by HPLC (Black: control leaf with cut petiole in distilled water and grey: tobacco leaf treated with Put solution 6 mM for 16 h). Samples of tissue were similar between control and treatment (~ 100 mg).
