# Supplementary material for: Evidence That Putrescine Modulates the Higher Plant Photosynthetic Proton Circuit
Source: PLoS One. 2012 Jan 12;7(1):e29864. doi: 10.1371/journal.pone.0029864 (PMC3257247; doi:10.1371/journal.pone.0029864)
Supplement: Figure S3 — Effects on Δψ/ pmf and PSII photochemical efficiency of infiltration of leaves with water. (DOC) [file pone.0029864.s003.doc]

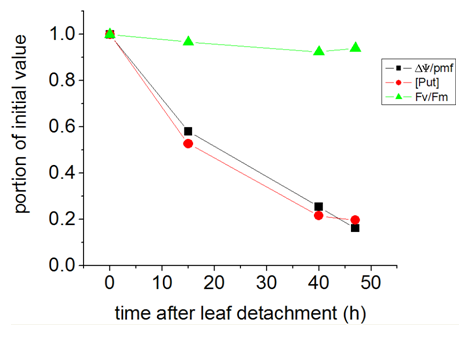


**Figure S3**. **Effects on Δψ/*pmf* and PSII photochemical efficiency of infiltration of leaves with water.** Tobacco leaf petioles were cut under 50 ml of distilled water results in a gradual decline of Δψ/*pmf*. At the same time the endogenous titer of Put declines whereas Fv/Fm is only marginally affected. The initial values were 0.43, 256 nmolgFW^-1^ and 0.78 for Δψ/*pmf*, [Put] and Fv/Fm respectively.
