# Supplementary material for: Evidence That Putrescine Modulates the Higher Plant Photosynthetic Proton Circuit
Source: PLoS One. 2012 Jan 12;7(1):e29864. doi: 10.1371/journal.pone.0029864 (PMC3257247; doi:10.1371/journal.pone.0029864)
Supplement: Figure S4 — Effect of putrescine titre on the dependence of energy-dependent antenna down-regulation (qE) on the ΔpH component of light-induced pmf . (DOC) [file pone.0029864.s004.doc]

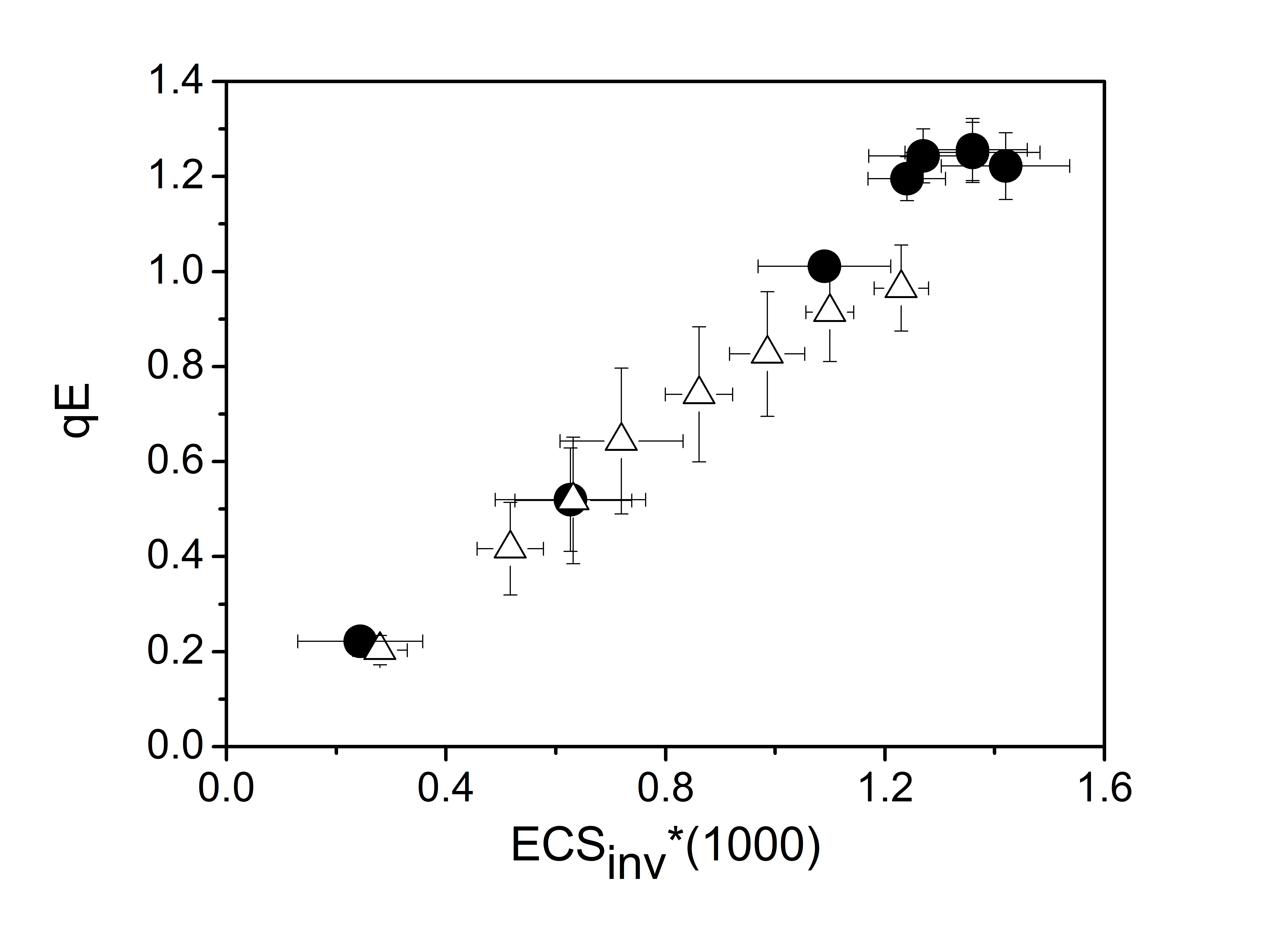


Figure S4. Effect of Put titre on the dependence of energy-dependent antenna down-regulation (q_E_) on the ΔpH component of light-induced *pmf*. The ΔpH component of light-induced *pmf* was estimated by the inverted ECS signal parameter (ECS_inv_). Tobacco leaves with high (open symbols) and low (closed symbols) Put titer were measured under steady state photosynthetic conditions with light intensities from 64 to 678 μmol of photons m^-2^s^-1^. Put increases the electric portion of *pmf* while antenna regulation by the ΔpH seems unaffected under these experimental conditions. Bars denote standard error for n= 4.
