# Supplementary material for: Evidence That Putrescine Modulates the Higher Plant Photosynthetic Proton Circuit
Source: PLoS One. 2012 Jan 12;7(1):e29864. doi: 10.1371/journal.pone.0029864 (PMC3257247; doi:10.1371/journal.pone.0029864)
Supplement: Figure S5 — Energy-dependent antenna down-regulation (qE) as a function of total light-induced pmf . (DOC) [file pone.0029864.s005.doc]

| A | 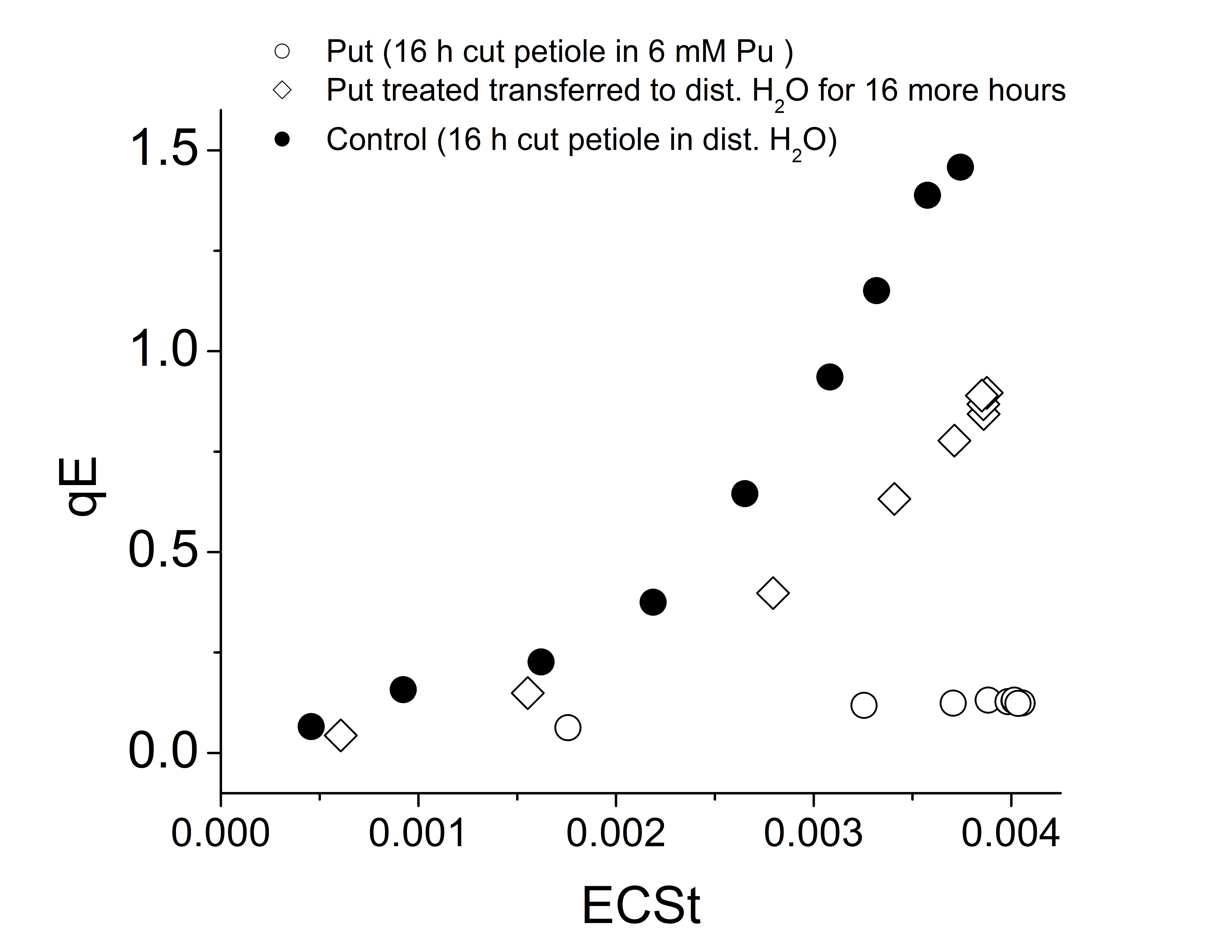 |
| --- | --- |
| B | 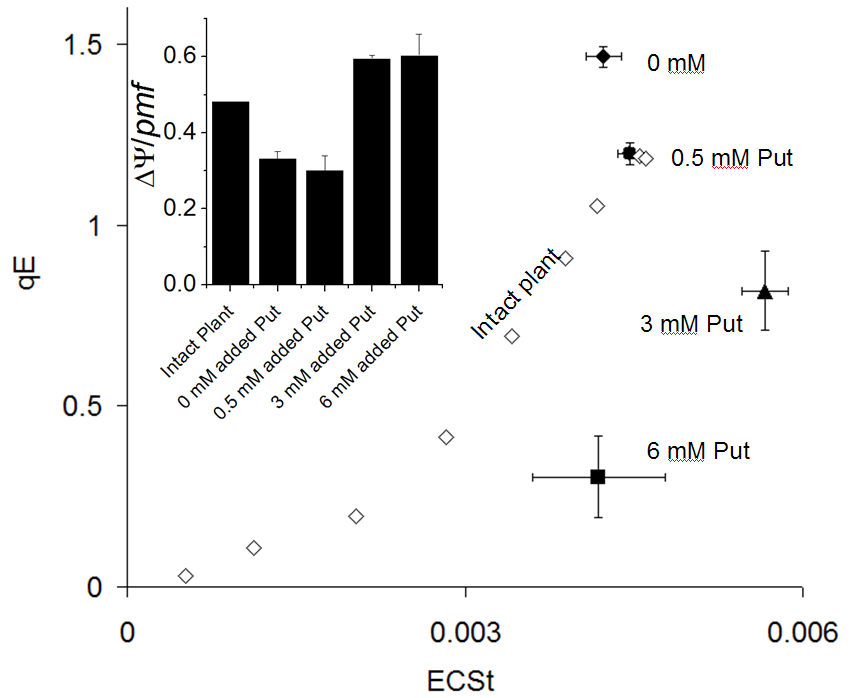 |

**Figure S5**. **Energy-dependent antenna down-regulation (q_E_) as a function of total light-induced *pmf.*** Tobacco leaves infiltrated through petiole with Put for 16 h (open circles) were measured under steady state photosynthetic conditions with light intensities from 64 to 678 μmol of photons m^-2^s^-1^ and show an almost complete inhibition of q_E_ activation at any light induced *pmf* tested (indications of a strongly buffered lumen pH). Untreated controls (detached leaf with petiole in distilled water) show normal activation of q_E_ (closed symbols). This striking effect is reversible to significant extent upon simple transfer of the leaf petiole in distilled water and further incubation for 16 h (open diamonds). The data in the panel A are from a single experiments illustrating the flexibility of the system. Similar dramatic effects were recorded with 3 independent experiments at a single light intensity and different doses of Put (0.5 mM, 3 mM and 6 mM Put) (panel B). This lower sensitivity was consistent with an increased Δψ/*pmf* (inset panel B). Bars denote standard error from 3 independent measurements while open diamonds correspond to the initial conditions (tobacco plant before leaf detachment).
