# Supplementary material for: Evidence That Putrescine Modulates the Higher Plant Photosynthetic Proton Circuit
Source: PLoS One. 2012 Jan 12;7(1):e29864. doi: 10.1371/journal.pone.0029864 (PMC3257247; doi:10.1371/journal.pone.0029864)
Supplement: Figure S6 — Effects of putrescine titre on the apparent proton conductivity of the ATP synthase (gH+). (DOC) [file pone.0029864.s006.doc]

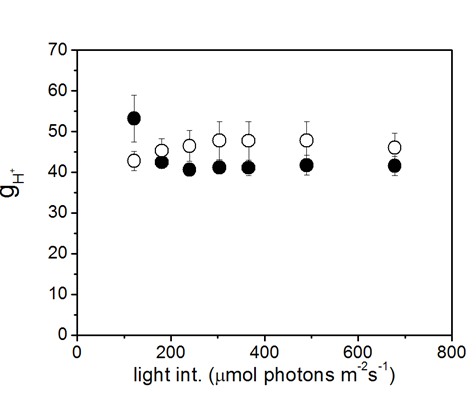


**Figure S6.** **Effects of Put titre on the apparent proton conductivity of the ATP synthase (g_H_^+^).** Estimates of g_H_^+^ for tobacco leaves infiltrated with 3 mM (open symbols) and 0 mM (closed symbols) Put titer were obtained by taking the inverse of the time constant for ECS decay during a 500-ms dark perturbation of steady-state conditions. Error bar denote SE for n= 4.
